# High levels of human infection with *Trypanosoma cruzi* associated with the domestic density of infected vectors and hosts in a rural area of northeastern Argentina

**DOI:** 10.1186/s13071-018-3069-0

**Published:** 2018-08-30

**Authors:** Marta Victoria Cardinal, Paula Andrea Sartor, María Sol Gaspe, Gustavo Fabián Enriquez, Ivana Colaianni, Ricardo Esteban Gürtler

**Affiliations:** 1 Universidad de Buenos Aires. Facultad de Ciencias Exactas y Naturales, Laboratory of Eco-Epidemiology, Ciudad Universitaria, C1428EHA Buenos Aires, Argentina; 2 Consejo Nacional de Investigaciones Científicas y Técnicas-Universidad de Buenos Aires. Instituto de Ecología, Genética y Evolución de Buenos Aires (IEGEBA), Ciudad Universitaria, C1428EHA Buenos Aires, Argentina; 3 Present address: Departamento de Control de Enfermedades Transmitidas por Vectores, Ministerio de Salud del Chaco, Resistencia, Chaco, Argentina, Universidad Nacional del Nordeste, Resistencia, Chaco, Argentina; 4 Present address: Hospital Santojanni, Ciudad de Buenos Aires, Argentina

**Keywords:** Gran Chaco, Seroprevalence, Chagas disease, *Triatoma infestans*, Dogs, Cats, Eco-epidemiology

## Abstract

**Background:**

Insecticide spraying campaigns designed to suppress the principal vectors of the Chagas disease usually lack an active surveillance system that copes with house reinvasion. Following an insecticide campaign with no subsequent surveillance over a 12-year period, we implemented a longitudinal intervention programme including periodic surveys for *Triatoma infestans*, full-coverage house spraying with insecticides, and selective control in a well-defined rural area of the Argentinean Chaco inhabited by Creoles and one indigenous group (Qom). Here, we conducted a cross-sectional study and report the age-specific seroprevalence of human *T. cruzi* infection by group, and examine the association between human infection, the onset of the intervention, the relative density of infected domestic bugs, and the household number of infected people, dogs, or cats.

**Results:**

The seroprevalence of infection among 691 residents examined was 39.8% and increased steadily with age, reaching 53–70% in those older than 20 years. The mean annual force of infection was 2.5 per 100 person-years (95% CI: 1.8–3.3%). Infection in children younger than 16 years born before the intervention programme was two to four times higher in houses with infected *T. infestans* than in houses without them and was six times higher when there were both infected dogs or cats and bugs than when they were absent. The model-averaged estimate of the intervention effect suggests that the odds of seropositivity were about nine times smaller for those born after the onset of the intervention than for those born before it, regardless of ethnic background, age, gender, household wealth, and cohabitation with *T. cruzi*-infected vectors or human hosts. Human infection was also closely associated with the baseline abundance of infected domestic triatomines and the number of infected cohabitants. Two of 43 children born after interventions were *T. cruzi*-seropositive; since their mothers were seropositive and both resided in apparently uninfested houses they were attributed to vertical transmission. Alternatively, these cases could be due to non-local vector-borne transmission.

**Conclusions:**

Our study reveals high levels of human infection with *T. cruzi* in the Argentinean Chaco, and the immediate impact of sustained vector surveillance and selective control actions on transmission.

**Electronic supplementary material:**

The online version of this article (10.1186/s13071-018-3069-0) contains supplementary material, which is available to authorized users.

## Background

Chagas disease remains a major cause of disability and premature death in the Americas [1]. Argentina leads the continental toll in the number of *Trypanosoma cruzi*-infected people according to recent estimates [2]. *Triatoma infestans*, the main domestic vector, has since 1991 been the target of a regional effort to eliminate the incidence of vector- and blood-borne *T. cruzi* infection in humans. This intergovernmental programme, denominated the Southern Cone Initiative, was mainly based on house spraying with pyrethroid insecticides and screening of blood donors [3, 4]. Sustained control actions reduced the abundance and geographical range of *T. infestans* and the incidence and prevalence of human infection with *T. cruzi* in several countries but failed to eliminate the vector from the Gran Chaco ecoregion, a sparsely populated plain extending over large sections of Argentina, Bolivia and Paraguay [5, 6]. Lack of continuity and contiguity of vector control actions allowed the resurgence of domestic vector-mediated transmission in the Argentinean and Bolivian Chaco and Peru [7–9], for example. In the dry Argentinean Chaco, sustained and supervised control actions suppressed vector-borne transmission whereas pulsed, unsupervised insecticide sprays left pockets of residual transmission [10–12]. Renewed transmission to humans was detected as early as 2–3 years after domestic reinfestation occurred and was preceded by domestic dog infections [10, 13].

A high seroprevalence of *T. cruzi* infection in indigenous communities has been reported in Argentina and the Americas [14–19]. Indigenous communities, Creoles and immigrant descendants inhabiting the rural Gran Chaco in many instances can be considered neglected populations facing limited access to health care services, electricity and safe water, exacerbated by remoteness and lack of infrastructure (i.e. dirt roads). Strong heterogeneities in the distribution of infection with *T. cruzi* among domestic triatomines, dogs and cats were revealed in a well-defined rural area of Pampa del Indio, northeastern Argentina [20]. Overall, *T. cruzi* infection was found in 26.0–28.7% of domestic *T. infestans*, dogs and cats, with large variations at household, village, and ethnic group levels [20]. Qom households (the only indigenous group present in the study area) were under a higher risk of bug, and dog infection than Creole households, which combined with a mathematical model of *T. cruzi* transmission [21] suggested that human infection would occur more frequently in Qom households. Based on these results and on evidence showing that indigenous communities in the Chaco are poverty-stricken and usually display substantial *T. cruzi* infection rates and more impoverished health conditions than local Creoles (e.g. [16, 18, 20]), our initial hypothesis was that Qom people would be at higher risk of infection.

This study is part of a longitudinal intervention programme on the eco-epidemiology and control of Chagas disease in Pampa del Indio, which included strategies to improve the access to diagnosis and treatment in rural settings [22]. Here we report the age-specific seroprevalence of human *T. cruzi* infection by ethnic group and examined the association between human infection and the onset of vector control interventions, the relative density of infected domestic bugs, and the household number of infected dogs or cats. The occurrence of *T. cruzi* infection in children ≤ 15 years of age is taken as an indicator of recent transmission [23, 24]. The study area had experienced two waves of vector control actions: the first, a government-supported, insecticide spraying campaign (with no subsequent surveillance) conducted 12 years before the second, prolonged wave (i.e. the longitudinal intervention programme), comprising full-coverage house spraying with insecticides, periodic vector surveys, and selective control [25–27]. Based on empirical and theoretical evidence [7, 12, 20, 21, 24], we hypothesized that human infection is strongly aggregated at household and village levels, increases monotonically with the relative abundance of infected domestic triatomines before interventions, and would be closely associated with the household presence of dogs and cats infected with *T. cruzi*, especially among children born before the intervention programme.

## Methods

### Study area

Fieldwork took place in a 450 km^2^ rural section of Pampa del Indio municipality, Chaco Province, Argentina, which encompassed 353 households grouped in 13 villages (10 de Mayo, 3 Lagunas, Campo Los Toros, Colonia Ombú, El Salvaje, Fortín Brown, La Herradura, La Loma, Las Bravas, Las Chuñas, Los Ciervos, Santa Rita and Santos Lugares). The study area, denominated Area I, has been described previously [20, 26]. A census of the local human population carried out in November 2011 enumerated a total of 1253 persons: 343 (27.4%) Qom and 880 (70.2%) Creole residents, 18 mixed ethnic backgrounds (1.4%), and 12 (1.0%) had missing data for the ethnic group. The head of each household responded to a questionnaire on socio-economic aspects, health-related practices and previous results of Chagas disease serodiagnosis. The last house spraying with insecticides conducted by vector control programmes before our longitudinal intervention programme had taken place in 1996, except for a few insecticidal treatments performed by local hospital personnel in 2006. Key events described below are depicted in a timeline (Additional file 1).

### Study design

We conducted two cross-sectional serosurveys aimed at complete enrollment of residents. These surveys were preceded by information, education, and communication workshops consisting of six meetings hosted at the closest schools or primary healthcare centres in which the local communities actively participated in survey planning [22]. All households in the study communities were invited to participate.

### Vector surveys

Following a baseline survey of house infestation, a community-wide house spraying with pyrethroid insecticide was conducted in November-December 2007 [26]. All existing houses were monitored for infestation every 4–7 months during the following three years and annually after that. Houses were selectively sprayed with insecticides if found positive for *T. infestans* [25]. A detailed georeferenced database of triatomine occurrence over the follow-up was built. Domestic infestations remained below 5% during the follow-up [25], and infested domiciles exhibited very low bug abundances [28].

### Domestic dogs and cats

A demographic and sero-parasitological survey targeting all domestic dogs and cats residing in 173 households from seven contiguous villages (10 de Mayo, Campo Los Toros, El Salvaje, La Loma, Las Chuñas, Los Ciervos and Santos Lugares) was conducted in August-December 2008 [20]. Dog and cat sera were tested for antibodies to *T. cruzi* using an indirect hemagglutination assay (IHA) following the manufacturer’s instructions (Wiener Laboratories S.A.I.C., Buenos Aires, Argentina) and an in-house ELISA. Serologically discordant sera were tested with IFAT (Ififluor Parasitest Chagas, Laboratorio IFI, Buenos Aires, Argentina) [20].

### Serosurvey

People eligible for the serosurvey were residents older than nine months of age who provided informed written consent; parents or guardians of children younger than 18 years of age provided consent for them. Venipuncture drew a blood sample of 3 ml (for children of 1–2 years of age) or 5–7 ml (older individuals) from each participating individual in October-November 2010 or January 2011. Each serum was separated after centrifugation at 3000× *rpm* for 15 min, allocated in triplicate vials and preserved at -20 °C. Another blood aliquot (1.5–3 ml) was immediately mixed with an equal volume of guanidine hydrochloride 6 M, EDTA 0.2 M buffer (pH 8.0) and stored at 4 °C for future reference.

### Serodiagnosis

Anti-*T. cruzi* antibodies were detected using two enzyme-linked immunosorbent assays (ELISA) including either semipurified fractions of epimastigote lysate or recombinant antigens (Chagatest, Wiener, and ELISA Rec V3.0, Wiener, respectively). Each serum was assayed in duplicates as described elsewhere [22]. A total of 715 serum samples were assayed at the Laboratory of Eco-Epidemiology, Buenos Aires, whereas 33 samples were assayed at the local hospital in Pampa del Indio. All tests were performed by the same persons (PAS and IC). Serologically discordant cases were tested by an indirect immunofluorescence antibody test (IFAT) (Ififluor Parasitest Chagas, Laboratorio IFI, Buenos Aires, Argentina). Serum samples reactive for at least two assays were considered seropositive for *T. cruzi*. Following guidelines issued by the federal government, *T. cruzi*-infected people ≤ 18 years of age were offered etiological treatment with benznidazole. Implementation of treatment delivery, follow-up and effects have been reported elsewhere [22].

### Data analysis

This manuscript complies with the STROBE checklist (Additional file 2: Table S1). Household data on human, dog, and cat infection were merged into the triatomine georeferenced database. Cohen’s kappa coefficient measured the degree of agreement between the serological tests employed (i.e. lysate ELISA, recombinant ELISA, and IFAT). Agresti-Coull 95% confidence intervals were calculated for seroprevalence rate [29]. Of 748 persons examined for infection, 57 resided in neighbouring localities outside of the study area and were excluded from current analyses. A mixed ethnic group was not included in the comparison of seroprevalence rates between subpopulation groups (Creole and Qom) because of its small sample size. The study villages were grouped according to distances and ethnic background. The missing age of one patient was estimated at 65 years of age on the basis of his eldest offspring. Statistical analyses were run in R (version 3.3.1) [30].

The best-fitting model of human infection was identified using an information-theoretic approach following the strategy outlined by Burnham & Anderson [31]. A random-intercept multiple logistic regression was fitted to a model that included all the explanatory variables (see below) using the *glmer* function implemented in the *lme4* package [32]. The package *MuMIn* [33] was used to dredge the full model considering all possible additive combinations of covariates and compute second-order Akaike’s information criterion corrected for small samples (AIC_c_), the difference between AIC_c_ and the lowest AIC_c_-scored model (∆AIC_c_), and model probabilities (i.e. Akaike weights) for each of the possible models. Models that differed in ≤ 2 AIC_c_ from the best-fitting model were considered the top models. The main risk factors were identified by relying on the relative importance (RI) of each explanatory variable and the size, sign and uncertainty of the coefficients. The overall quality of the fitted logistic regression models was assessed using the Hosmer-Lemeshow goodness-of-fit test using the package *ResourceSelection* [34]. We also calculated the H-index as a measure of the classification performance of the models [35] which considers misclassification costs, misclassifying a person as seronegative (which in practice means losing an opportunity of etiological treatment) was a more costly mistake than misclassifying a seronegative as seropositive. The H-index ranges from 0 to1 and allows comparisons of models across different datasets and classifiers. The H-index calculation was implemented using the *hmeasure* in the R software package [31].

Putative predictors of human infection were established a priori based on existing evidence [13, 20, 21, 24, 36, 37] on the major role of *T. infestans*-mediated transmission, including the density of infected bugs, exposure time, the occurrence and number of infected dogs and cats, number of infected co-inhabitants, and current hypotheses on potential effects of ethnic background. The random-intercept (i.e. random-effects) model allows for the fact that observations on human infection at the household level are not independent. The goat-equivalent index, used as a household-level surrogate for wealth, was computed considering the total number of livestock (cows, pigs and goats) and poultry owned by the household regarding relative goat biomass as described before [38]. Reference levels were being born before the onset of the intervention programme, the mean age, females, Creoles, the mean number of infected cohabitants, and the mean goat-equivalent index. Given that the total bug and infected-bug abundances were highly correlated (Pearson’s *r* = 0.85, *P* < 0.0001), both variables were subsumed into a new variable (relative infected-bug abundance per 15 minutes-person) categorized in four levels (no bugs collected; 0 infected bugs; 1–9 infected bugs; and ≥ 10 infected bugs) as described in [20]. However, in the final model, the coefficient CIs of the variable levels overlapped. Therefore, it was simplified in a 3-level variable (no bugs collected; no infected bugs; ≥ 1 infected bugs).

The first global model included a total of 573 people (residing in 142 households), who had no missing data in the study variables. Because house infestation data at baseline were missing for 65 persons examined for *T. cruzi* infection, they were excluded from regressions analyses. A total of 16 (36%) Qom and 18 (14%) Creole households were excluded for this reason; the proportion of households with missing data was significantly higher among Qom than Creole households (Fisher’s exact test, *P* = 0.004). The potential interaction between ethnicity and every other factor was investigated on a post hoc basis, adding terms one by one to each global model to avoid convergence problems; all interaction terms proved non-significant. Multicollinearity was assessed by using the “collin” command in Stata 12.1 (Stata Corp LP, USA). Mean VIF for the dataset including the intervention effect was 1.19. VIF values for each variable ranged between 1.02–1.40. The condition number was 5.6. These diagnostics showed there was no significant multicollinearity in the dataset.

Because the dog and cat infection survey comprised a subset of the villages, we ran a second model not shown here which included the household number of infected dogs and cats as a predictor, yielding a total of 336 humans and 86 houses with complete data for the study variables. However, the number of infected cohabitants, infected-bug abundance and the household number of infected dogs and cats were significantly and positively correlated (multicollinearity) in this dataset. Therefore, the joint effect of vector and domestic host infection on human infection prevalence was tentatively evaluated using a non-parametric Cochran-Mantel-Haenszel test.

The mean annual force of infection (λ) in humans was estimated retrospectively using a catalytic model [39]. This model assumes that the incidence of infection is constant over time and age-independent. The recovery rate was set to zero to represent the absence of serorecovery or specific chemotherapy. λ was estimated using Stata 15.0 (StataCorp. 2017. Stata Statistical Software: Release 15. College Station, TX: StataCorp LLC, USA).

## Results

A total of 691 persons were examined for infection, representing 55% of the enumerated population (*n* = 1253) and residents of 54% of the 360 inhabited houses. Creoles represented 69.8% of the examined population; thus, the ethnic composition in the dataset did not differ from the total population (Table 1). The main reasons for lack of participation were: transient absence from the study area (14%); unavailable to assist (11%); ignorance of the survey (9%); and other (3%) or unknown (58%) reasons; 2% refused venipuncture; 2% were uninterested; and 2% reported having a previous serodiagnostic result. The overall seroprevalence of *T. cruzi* was 39.8% (95% CI: 36.2–43.5%, Table 1). Highly concordant results between both ELISA assays were obtained: co-positivity was 95.0% and co-negativity 98.9% (Additional file 2: Table S2). The kappa coefficient between ELISA tests was 0.94 (95% CI: 0.92–0.97); between IFAT and recombinant ELISA 0.72 (95% CI: 0.49–0.95), and between IFAT and lysate ELISA 0.41 (95% CI: 0.16–0.66), showing very good, good, and moderate accordance, respectively.

**Table 1:** Seroprevalence of *T. cruzi* infection and demographic attributes according to the village of residence in Area I of Pampa del Indio, Chaco

Village group	No. of houses	% Creoles	% born after the intervention programme	% males	% ≤ 15 years-old	% houses with ≥ 1 infected person	No. of examined inhabitants	Human seroprevalence	
								%	95% CI
10 de Mayo	28	15.6	3.1	43.8	51.0	67.9	96	47.9	38.2–57.8
SV-LL-BV-CV	39	100.0	5.6	47.8	52.2	74.4	161	42.2	34.9–50.0
3L-CO-H	19	19.4	11.1	45.8	50.0	73.7	72	44.4	33.5–55.9
CT-CHU-RI	52	72.4	7.5	49.1	52.6	73.1	228	39.9	33.8–46.4
Fortín Brown	16	87.7	3.5	40.4	54.4	56.3	57	22.8	13.7–35.3
LUG	22	100.0	5.2	55.8	48.1	68.2	77	32.5	23.0–43.6
Total	176	69.8	6.2	47.8	51.7	71.0	691	39.8	36.2–43.5

*Abbreviations: SV* El Salvaje, *LL* La Loma, *BV* Las Bravas, *CV* Los Ciervos, *3L* Tres Lagunas, *CO* Colonia Ombú, *H* La Herradura, *CT* Campo los Toros, *CHU* Las Chuñas, *RI* Santa Rita, *LUG* Santos Lugares

The median age of the study population was 15 years (range, 11 months - 81 years) and 47.8% were males. The majority (60%) of the examined population was native to the study area; 12% were born elsewhere, and 28% had missing data for this variable. Qom residents were distributed unevenly among the study villages whereas the percentage of males and people under 16 years of age was similar among them (Table 1). Creole households had a 25× higher median goat-equivalent index (median, interquartile range: 55.6, 10.3–190.7) than Qom households (2.2, 0.3–27.8).

At least one seropositive person was found in 71.0% of the 176 study households (Table 1). The distribution of the numbers of cases of *T. cruzi* infection in households marginally fitted a negative binomial distribution (goodness-of-fit *χ*^2^ = 7.3, *df* = 3, *P* = 0.06); the distribution parameter (k = 3.2; SE = 0.8) suggested that cases were not strongly aggregated. No infected resident was found in 29% of all households whereas 34%, 17% and 20% of them harboured 1, 2 and ≥ 3 infected residents, respectively. The seroprevalence of *T. cruzi* differed among villages and was two times higher in 10 de Mayo (47.9%) than in Fortín Brown (22.8%) (Table 1).

**Fig. 1:**
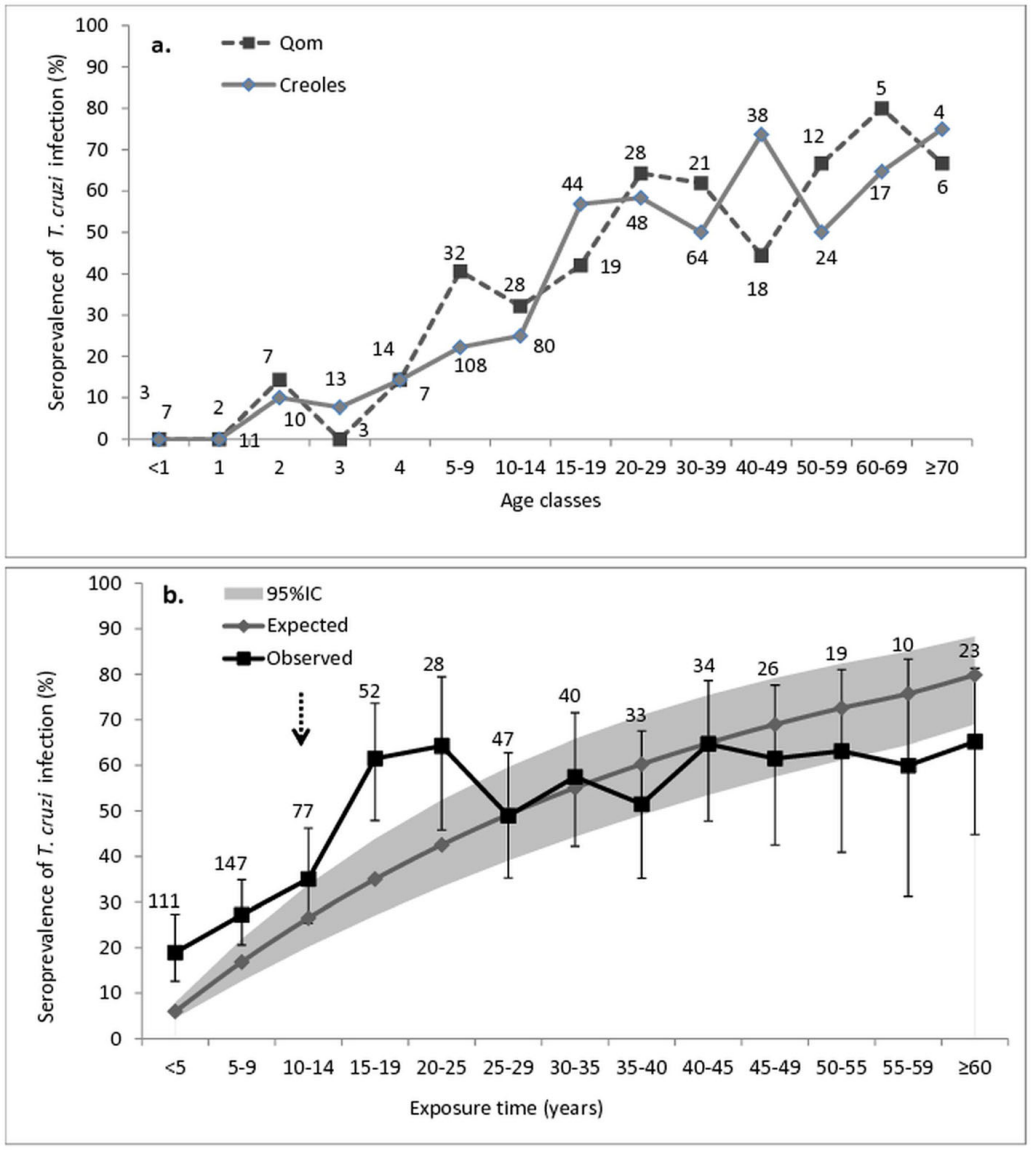
Age-specific prevalence of seropositivity for *Trypanosoma cruzi* infection in Area I residents by ethnic group (**a**) and observed (black) and predicted (grey line) seroprevalence according to an irreversible catalytic model with a constant force of infection (**b**) in Pampa del Indio, Chaco. The arrow indicates when the last community-wide insecticide spraying was conducted. Numbers close to data points represent the numbers of humans examined for infection; bars are 95% CI. This figure excludes 18 people from a mixed ethnic background. Multimodel inference analysis provided little support for including an ethnic group in the best models

The seroprevalence of *T. cruzi* steadily increased with age both among Creoles and Qom residents (univariate odds ratio, OR = 1.97; 95% CI: 1.64–2.37) (Fig. 1a) (Table 4). No case of *T. cruzi* infection was detected among 25 children ≤ 1-year-old examined. Seroprevalence then increased to nearly 10% in children aged up to 5 years, rose steeply to 51% in young adults aged 15–19 years, and fluctuated between 53–70% in older adults (Fig. 1a, b). The mean annual force of infection was 2.5 per 100 person-years (95% CI: 1.8–3.3%) (*n* = 13 age groups). The predicted age-specific proportions of infected individuals departed significantly from observations (i.e. were outside the 95% CI) in young people up to 25 years of age and tended to underestimate the observed seroprevalence at older ages (Fig. 1b).

Children born after the onset of the intervention programme (up to exactly 3 years-old) exhibited a significantly lower seroprevalence to *T. cruzi* (4.7%, mean age = 1.3 years, *n* = 43) than residents ≤ 5 years of age born before it (23.0%, mean age = 4.1 years, *n* = 61; Fisher’s exact test, *P* = 0.01). The two *T. cruzi* -seropositive children (one Creole girl and one Qom boy) born after interventions were born to *T. cruzi*-seropositive mothers. Both cases resided in houses with no infected bug detected during the post-intervention follow-up (Table 3), although both houses had harboured *T. cruzi-*infected *T. infestans* at baseline. The seropositive girl cohabited with four infected dogs and six infected people, whereas the boy cohabited with seven infected people (the serological status of dogs and cats was not recorded). Both houses remained uninfested during four and six vector surveys, respectively, after both children were born (Table 2).

**Table 2:** Seroprevalence of *Trypanosoma cruzi* in children born after the intervention programme and observed exposure to domestic, peridomestic and *T. cruzi*-infected *Triatoma infestans* over 2008–2010, Pampa del Indio, Chaco

Factor	% seropositive (no. examined)
Observed domestic infestation	
No	5.1 (39)
Yes	0.0 (4)
Observed peridomestic infestation	
No	5.4 (37)
Yes	0.0 (6)
Presence of infected bugs	
No	4.8 (42)
Yes	0.0 (1)

**Fig. 2:**
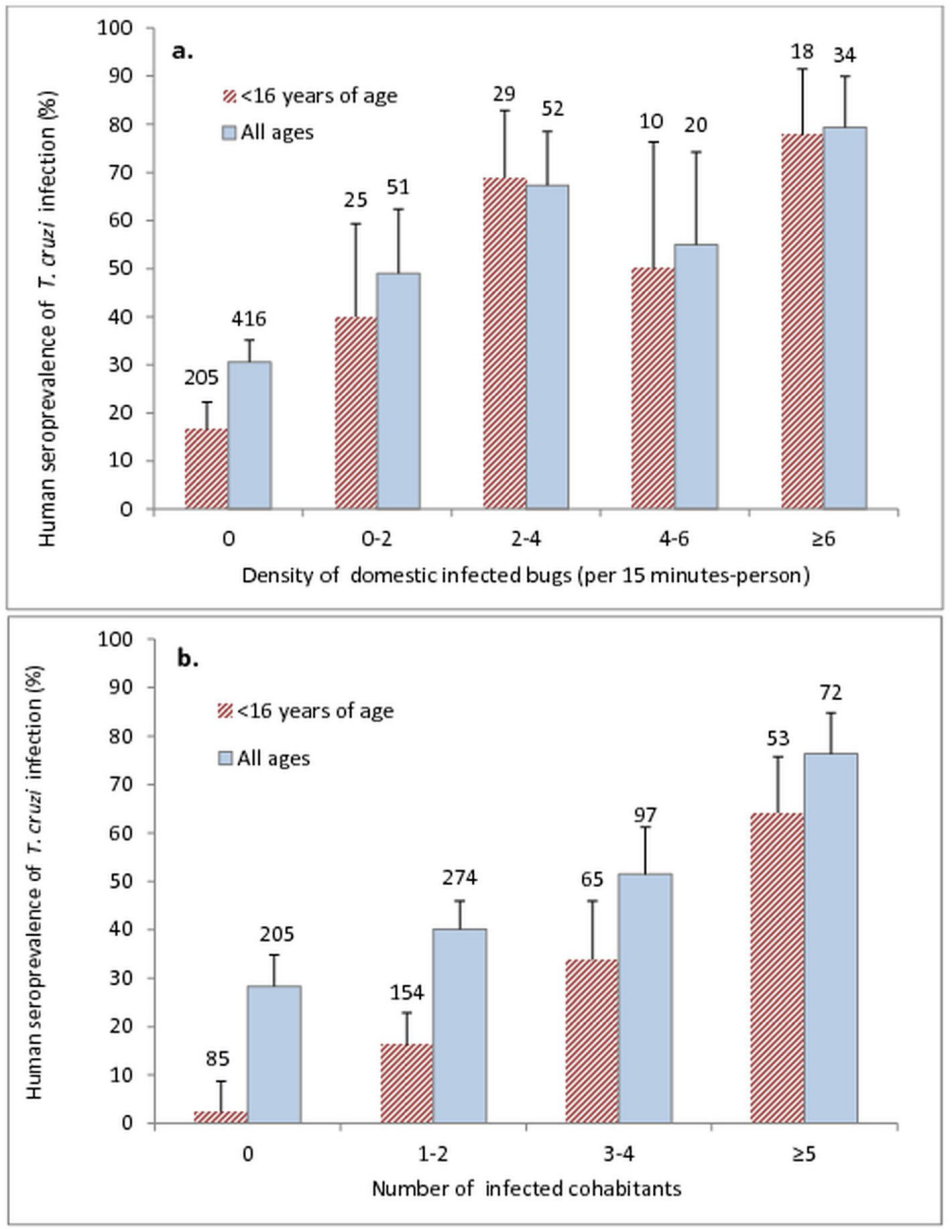
Relative abundance of *Trypanosoma cruzi-*infected domestic *Triatoma infestans* (**a**) and some infected cohabitants (**b**) and age-specific prevalence of seropositivity to *T. cruzi* in people born before the onset of the intervention programme in Area I of Pampa del Indio, Chaco. Numbers above bars indicate the number of people examined for infection. This figure excludes 118 people with no house infestation data

*Trypanosoma cruzi* infection in children younger than 16 years of age born before the intervention programme was 2–4 times higher in houses with infected *T. infestans* than in houses without them and was 6 times higher in houses harboring infected dogs or cats and infected bugs than in houses with no infected host or infected vector (Table 3). A significant effect of bug infection on human *T. cruzi* seroprevalence was observed for all age groups combined (Cochran-Mantel-Haenszel *χ*^2^ = 23.5, *df* = 1, *P* < 0.0001) (OR = 3.06, 95% CI: 1.70–5.52 for houses with infected dogs and cats; OR = 2.03, 95% CI: 1.02–4.04 for houses with non-infected dogs and cats; OR = 2.21, 95% CI: 1.20–4.07 for houses with no data on dog and cat infection status) and for children younger than 16 years of age born before the onset of interventions (Cochran-Mantel-Haenszel *χ*^2^ = 38.2, *df* = 1, *P* < 0.0001), for each level of dog or cat infection status (OR = 12.69, 95% CI: 3.91–41.17 for houses with infected dogs and cats; OR = 3.04, 95% CI: 1.05–8.77 for houses with non-infected dogs and cats; OR = 6.21, 95% CI: 2.22–17.35 for houses with no data on dog and cat infection status) (Table 3). A significant effect of dog or cat infection on human *T. cruzi* seroprevalence was observed for all age groups combined for each level of bug infection status (Cochran-Mantel-Haenszel *χ*^2^ = 4.6, *df* = 1, *P* = 0.034) (common OR = 1.66, 95% CI: 1.07–2.59), but not for people born before the intervention programme (Table 3). Univariate analyses revealed no significant association between *T. cruzi* infection and the goat-equivalent index, ethnic group or gender (Table 4). The multimodel inference framework identified six top models of human infection (Table 5). *T. cruzi* infection in residents was significantly and positively associated with age, the baseline abundance of infected domestic *T. infestans*, and the household number of infected cohabitants; and was negatively associated with being born after the onset of the intervention programme (Fig. 2) (Tables 4, 5, and Additional file 2: Table S3) (Hosmer-Lemeshow goodness-of-fit test *χ*^2^ = 13.6, *df* = 8, *P* = 0.09). The intervention programme was associated with a sharp decline in infection risk. Specifically, the adjusted, model-averaged estimate of the intervention effect (beta = -2.2, SE 0.8) suggests that the odds of seropositivity was 9.2 (95% CI: 1.9–45.2) times smaller for those born after the onset of the intervention than for those born before it, regardless of ethnic background, age, gender, household wealth, and cohabitation with *T. cruzi*-infected vectors or human hosts (Table 4 and Additional file 2: Table S3). The H-index was 0.36, indicating a fair performance of the model, the area under the ROC curve was 0.81, and the sensitivity and specificity of the model were 0.54 and 0.82, respectively. Gender, ethnic group and the goat-equivalent index had low relative importance (Tables 4, 5). The effect of the household as a random variable was virtually nil, and no difference was found between models that included it or not (Log-likelihood ratio test, *P* = 1), suggesting the variables in the model may have removed any excess variation due to household clustering effects.

**Table 3:** Distribution of household seroreactivity to *T. cruzi* according to the infection status of dogs or cats and *T. infestans* in Area I of Pampa del Indio, Chaco

Dog or cat infection status	Vector infection status	No. of houses	All people		Born before the intervention programme			
			All ages		> 15 years of age		< 16 years of age	
			*n*	%^a^	*n*	%^a^	*n*	%^a^
Infected	Infected	23	100	57.0	48	62.5	47	55.3
	Non-infected	23	96	30.2	44	56.8	45	8.9
	Subtotal	46	196	43.9	92	59.8	92	32.6
Non-infected	Infected	10	44	52.3	24	58.3	20	45.0
	Non-infected	34	134	35.1	62	53.2	66	21.2
	Subtotal	44	178	39.3	86	54.7	86	26.7
No data	Infected	12	53	49.1	22	63.6	25	44.0
	Non-infected^b^	61	214	30.4	109	50.5	89	11.2
	No data	15	50	56.0	26	88.5	20	35.0
	Subtotal	88	317	37.5	157	58.6	134	20.9
Total		178	691	39.8	335	57.9	312	26.0

^a^% seropositive

^b^Includes one house with no dogs or cats

*Abbreviation*: *n* number of persons examined

**Table 4:** Univariate and multivariate (model-averaged) odds ratio of potential risk factors for human infection with *T. cruzi* in residents of Area I of Pampa del Indio, Chaco

Predictor	Mean (SD)	Univariate analysis		Multivariate analysis (*n* = 573)	
		OR	95% CI	OR	95% CI
Born after the onset of the intervention					
No		1.00	–	1.00	–
Yes		0.08	0.01–027	0.11	0.02–0.52
Age	22.3 (18.4)	1.97	1.64–2.37	2.54	2.01–3.20
Goat-equivalent index	9.5 (23.2)	0.85	0.70–1.02	0.89	0.73–1.09
Ethnic group					
Creole		1.00	–	1.00	–
Qom		1.32	0.89–1.94	0.65	0.39–1.09
Gender					
Female		1.00	–	1.00	–
Male		1.15	0.82–1.61	1.14	0.77–1.71
Infected-bug abundance					
No bugs		1.00	–	1.00	–
No infected bugs		0.98	0.62–1.53	1.17	0.70–1.97
≥ 1 infected bugs		3.75	2.50–5.67	2.88	1.68–4.94
No. of cohabiting infected persons	1.8 (1.9)	1.91	1.60–2.31	2.29	1.80–2.92
Number of cohabiting infected dogs or cats					
0		1.00	–	–	–
1		0.63	0.36–1.11	–	–
≥ 2		1.70	1.02–2.84	–	–

*Abbreviations*: *CI* confidence interval, *OR* odds ratio

**Table 5:** Multi-model assessment of factors associated with *T. cruzi* infection in humans (complete dataset, *n* = 573) in Area I of Pampa del Indio, Chaco

Top models		*df*	Variables analyzed^a^						Model fit^b^			
			1-Ethnicity	2-Infected- bug abundance	3-No. of infected cohabitants	4-Gender	5-Age	6-Goat- equivalent index	7-Born after the onset of the intervention programme	logLik	∆AIC_ci_	ω_i_
1	12357	8	×	×	×	–	×	–	×	-293.7	0.00	0.23
2	123567	9	×	×	×	–	×	×	×	-292.9	0.46	0.18
3	2357	7	–	×	×	–	×	–	×	-294.9	0.47	0.18
4	123457	9	×	×	×	×	×	–	×	-293.4	1.61	0.10
5	23567	8	–	×	×	–	×	×	×	-294.5	1.62	0.10
6	23457	8	–	×	×	×	×	–	×	-294.7	2.01	0.08
Relative importance			0.59	1.00	1.00	0.31	1.00	0.41	0.99	–	–	–

^a^Variables (see text for details): Ethnicity, ethnic background of each person (Qom *versus* Creole or mixed background); relative infected-bug abundance, 3 levels; number of *T. cruzi*-infected persons each person cohabited with; gender; age (in years); goat-equivalent index per 10 goats; being born after the onset of the intervention programme (2 levels)

^b^∆AIC_ci_ = AIC_ci_ − AIC_cmin;_ ω_i_ = exp(−1/2 ∆AIC_ci_)/S exp (−1/2 ∆AIC_ci_); AIC_c=_ Akaike’s information criterion corrected for small samples

*Key*: ×, variable included in model; –, variable not included in model

## Discussion

Our study reveals high levels of human infection with *T. cruzi* closely related to infected-bug abundance in a mostly rural area of the Argentinean Chaco with widely variable ethnic and demographic characteristics. The higher rates of seroprevalence in older people are the consequence of cumulative exposure to vector-mediated transmission over a lifetime, whereas infection among people younger than 16 years of age (23.2%) documents the recent occurrence of vector-borne transmission in the absence of sustained vector and disease surveillance. Conversely, residents born after the onset of the intervention programme were marginally exposed to *T. infestans* (infected or not) and had 9.2 smaller odds of seropositivity and a 4.9 (95% CI: 2.1–35.2) times lower seroprevalence than children less than five years of age born before interventions. Although the latter group had a shorter period of exposure to triatomines (i.e. a fraction of the differential risk between groups should be discounted), the vast difference attests to the tremendous impact of sustained vector control on parasite transmission. The high seroprevalence recorded is among the highest reported since the launching of the Southern Cone Initiative and exceeds the estimated national seroprevalence of *T. cruzi* (3.64%) [2] by a factor of 11. The serosurvey thus reveals the substantial burden of Chagas disease in the study population, which is unlikely to become visible in the context of limited access to health care services including serodiagnosis and treatment. These patterns may be familiar to high-risk settings of the Gran Chaco region where only sporadic vector control actions are implemented.

A relatively high predicted baseline probability of infection was found (32.3%, CI: 25.3–40.1%, intercept in Additional file 2: Table S3). This probability thus represents the estimated prevalence among Creole women of average age (22.3 years), living in an uninfested house, cohabiting with the average number (1.8) of infected cohabitants, exhibiting the average goat-equivalent index (9.5) and born before the onset of intervention. This high prevalence estimate could be because exposure to infestation or infected bugs was derived from a cross-sectional house infestation survey performed in 2007 before the spraying campaign. Therefore, the patients’ seropositivity status (which is the product of cumulative exposure times a surrogate of vectorial capacity) was related to a point infestation measure. Some patients who inhabited uninfested houses in 2007 had likely been exposed to triatomines in the past, and hence this would explain the relatively high predicted infection probability at baseline. Similarly, the number of infected cohabitants was derived from serodiagnostic surveys conducted over 2010–2011; therefore, this measure may not adequately represent the exposure linked to cohabiting with infected people in the past.

*Trypanosoma cruzi* infection steadily increased with age up to 20–29 years, with more than half of all people aged > 20 years seropositive to *T. cruzi*. The seroprevalence curve reflects a high force of infection acting at the onset of the intervention programme despite the insecticide campaign conducted 12 years before. Fast recovery of domestic transmission in the absence of vector surveillance has been widely documented in endemic areas where *T. infestans* is prevalent [7–9, 40]. More specifically, among people younger than 16 years of age, the odds of *T. cruzi* seropositivity was three to six times higher in houses harboring infected *T. infestans* at baseline than in houses without them and was 13 times higher in houses harboring *T. cruzi*-infected dogs, cats and infected *T. infestans* than in houses without them (Table 3). In the multivariate analysis, human infection was significantly associated with the household number of infected people and the baseline abundance of infected domestic bugs. A strong association between the relative abundance of infected domestic bugs and the seroprevalence of *T. cruzi* infection in domestic dogs was recorded previously [20]. These results support the occurrence of vector-borne transmission before the intervention programme and point to the significant contributions of chronically infected people jointly with domestic dogs or cats to the maintenance of domestic parasite transmission. Although chronic human infections are characterised by low parasitemia [41–43], their contribution to domestic transmission may be enhanced by regular human-vector contact rates [44].

A relevant result of this study refers to the relative density of *T. cruzi*-infected domestic *T. infestans* associated with human infection. People residing in houses harbouring as few as one infected domestic triatomine per unit effort exhibited 2.9-fold higher odds of seropositivity than people residing in houses harbouring no infected bug at baseline (Fig. 2). This suggests that if there is a threshold density of infected bugs needed to trigger domestic transmission, it would be very low [7, 45] and hard to reveal given the low sensitivity and imprecision of timed-manual searches for triatomines [25, 46–48]. Indeed, the detection of a single infected bug could be useful as an index of transmission risk. Taken together, these results support the promotion of zero tolerance to domestic infestations with *T. infestans*. Moreover, the association between infected bug abundance and the seroprevalence of infection both in humans (this study) and dogs [20], derived as cross-sectional estimates that result from a long-term process, suggest the observed infected bug abundance could be an index of transmission risk.

Transfusional and vertical transmission may also account for some of the observed human infections with *T. cruzi*. Blood donors in Argentina began to be regularly screened for *T. cruzi* antibodies in the 1960s, and full coverage may have been achieved by the 1980s [49]. In our study, 5% (*n* = 691) of the study population recalled having received a transfusion, which suggests that some of the older people may have been exposed to infected blood. Vertical transmission is a permanent source of new infections, especially in highly endemic areas where women in reproductive age both have high seroprevalence rates (54% in our study area) and high fertility. As the mother’s name of each examined person was not systematically recorded, maternal serostatus could not be accounted for in our multivariate analysis. The two infected children born after the intervention programme most likely were congenital cases that had not been detected by healthcare services, given their mothers were also seropositive and had not received etiological treatment and they resided in apparently uninfested houses. If manual searches for triatomines may generate 40% of false negative results [46], the cumulative probability that these two houses were infested given repeated manual searches with an adverse outcome ranges between 0.4–2.5%. Successive negative results are compatible with very low bug abundance. Given that infected bug abundance was highly correlated with overall bug abundance, taken together these results suggest the probability these two children were exposed to infected bugs was extremely low. In contrast, the average risk of vertical transmission from *T. cruzi*-seropositive mothers to newborns ranges from 1.9–9.1% [50–52]. Failure to diagnose congenital cases at birth or during routine controls over the first year of life translates into a lost opportunity for etiological treatment and cure. Implementing the diagnosis and treatment of congenital cases in remote rural areas of the Gran Chaco region remains an unresolved challenge. Alternatively, these two cases could be due to vector-borne transmission occurring in neighbouring villages under sporadic or no vector control actions (i.e. non-local transmission), as recorded for infected dogs elsewhere in the Argentinean Chaco [12]. Given that the history of travelling outside the study area is affected by recall bias and was not recorded, this transmission route cannot be ruled out.

Indigenous communities in the Chaco are poverty-stricken, and usually display substantial *T. cruzi* infection rates and poorer health conditions than local Creoles (e.g. [16, 18, 20]). In our study area, the median goat-equivalent index of Creole households was 25 times larger than Qom’s. However, when more proximal variables in the causal chain were included in the models (i.e. the household number of infected people and infected-bug abundance), we found little evidence supporting the inclusion of the ethnic group in the best model, which refutes our initial hypothesis. This result coincides with the lack of association between ethnic background and the prevalence of house infestation with *T. infestans* in Area I, which was at least partially related to the considerable variability in housing and socio-economic conditions within each ethnic group [26]. Qom households from a nearby rural section of Pampa del Indio (Area III) exhibited a lower, less variable goat-equivalent index [38] than that observed in Qom households from Area I. These heterogeneities within a single ethnic group and district point to more a complex system defying a simple stratification by ethnicity.

The present results are consistent with the critical role that domestic dogs and cats play as domestic reservoir hosts of *T. cruzi* throughout the Americas [13]. Their infection was closely and positively correlated with the density of infected *T. infestans* [20, 31, 33, 34] and with the relative odds of human infection with *T. cruzi* elsewhere [30]. Infected dogs and cats are important domestic sources of *T. cruzi* infection because of their high infectiousness to the vector [33, 53] and frequent occurrence as bloodmeal sources of *T. infestans* [33, 38]. In rural endemic areas, dogs and cats are usually unrestrained and may provide a link between domestic and sylvatic transmission cycles through predation of small sylvatic reservoirs (e.g. rodents, marsupials). Treatment of infected dogs that diminish their infectiousness or repellents that reduce dog-vector contact rates would exert a tremendous impact on domestic transmission risks [13].

Our study has both strengths and limitations. The detailed database, including nearly three years of frequent monitoring of house (re)infestation and the infection status of domestic dogs, cats and triatomines, enabled us to probe into the domestic transmission process. The fact that dogs and cats were examined for infection almost one year after the onset of the intervention programme implies that a fraction of the seropositive animals at baseline was lost for detection because of the fast turnover of rural dog and cat populations. Therefore, dog infection estimates most likely underestimated their actual status at baseline. A total of 76 (11%) people with a serodiagnostic result resided in houses built after the onset of the intervention programme and therefore lacked a pre-intervention measure of house infestation status and bug abundance, and 28% of the examined population had missing data for their place of origin. We were not able to trace back their previous place of residence to match their previous exposure to current infection status nor did we record travel history and frequency outside of the study area. Although the study population in Area I was more stable than in other Qom communities of Pampa del Indio [38], the high rates of household mobility and visitation for extended periods among the Qom suggest some of the existing infections may have been acquired in other districts lacking vector control actions. Although our study aimed at full coverage of the study communities, a fraction of the resident human population was not examined. Self-selection bias may lead to underestimating or overestimating the actual seroprevalence rates. If older people were under-represented in the sample or if infected people with a previous diagnosis refused to provide a blood sample, we would have underestimated the true seroprevalence of infection. Conversely, if infants and young children were lost for diagnosis or if uninfected people chose not to participate in the serosurvey, this would lead to overestimating the true seroprevalence. However, very few (0.8%) residents reported having a previous serodiagnosis and the achieved diagnostic coverage was relatively high (70.3%) among residents ≤ 18 years of age [22].

The large seroprevalence rate of *T. cruzi* (23.3%) among children born between the 1996 insecticide spraying campaign and the 2007 intervention programme can be mainly attributed to vector-borne transmission and reflects the consequences of not implementing effective vector surveillance and control. Vector-mediated transmission to domestic dogs, cats and humans were also documented in a scenario of pulsed vector control actions elsewhere in the dry Chaco region [12] and Peru [8]. In this context, rapid house reinvasion of *T. infestans* and the availability of long-lived, infected domestic hosts as blood sources for the bugs lead to the resurgence of domestic transmission. The continued interruption of domestic *T. cruzi* transmission demands sustained political commitment to assure long-term vector surveillance and adequate response actions, more so in low-density, remote settings as in the Chaco. Our results suggest a sharp decline of vector-mediated transmission risk, perhaps to the point of near-zero incidence in the local human population, associated with the onset of the intervention programme. Scaling up of systematic vector control interventions and enhancing access to serodiagnosis and etiological treatment [22] are crucial to diminish the burden of Chagas disease in the affected populations.

## Conclusions

We detected a widespread, high overall seroprevalence of human infection with *T. cruzi*, which exceeded 50% in residents aged ≥ 20 years of a well-defined human population of the Argentinean Chaco. Human seroprevalence was mainly associated with the baseline density of infected domestic bugs and the household number of infected cohabitants. Sustained vector surveillance and selective control actions were associated with the apparent interruption of vector-mediated transmission.

## Additional files

Additional file 1: Timeline of key events. (PDF 203 kb)

Additional file 2: **Table S1.** STROBE Statement - checklist of items that should be included in reports of cross-sectional studies. **Table S2.** Comparison among serological test results for *Trypanosoma cruzi* infection in Area I human residents of Pampa del Indio, Chaco. **Table S3.** Model-averaged coefficients of factors associated with *Trypanosoma cruzi* infection in humans born before the intervention programme in Pampa del Indio, Chaco. (DOCX 33 kb)

## References

[1] Hotez PJ (2014). The medical biochemistry of poverty and neglect. Mol Med..

[2] WHO (2015). Chagas disease in Latin America: an epidemiological update based on 2010 estimates. Wkly Epidemiol Rec..

[3] Schmunis GA, Zicker F, Moncayo A (1996). Interruption of Chagas’ disease transmission through vector elimination. Lancet..

[4] Silveira A. O controle da doença de Chagas nos países do Cone Sul da América: história de uma iniciativa internacional 1991–2001. In: Silveira AC, editor. El control de la enfermedad de Chagas en los países del Cono Sur de América. Historia de una iniciativa internacional 1991/2001, vol. 2002. Buenos Aires: PAHO. p. 15–44.

[5] Gürtler R (2009). Sustainability of vector control strategies in the Gran Chaco region: current challenges and possible approaches. Mem Inst Oswaldo Cruz..

[6] Tarleton RL, Reithinger R, Urbina JA, Kitron U, Gürtler RE (2007). The challenges of Chagas disease - grim outlook or glimmer of hope?. PLoS Med..

[7] Gürtler RE, Cecere MC, Lauricella MA, Petersen RM, Chuit R, Segura EL (2005). Incidence of *Trypanosoma cruzi* infection among children following domestic reinfestation after insecticide spraying in rural northwestern Argentina. Am J Trop Med Hyg..

[8] Delgado S, Castillo Neyra R, Quispe Machaca VR, Ancca Juárez J, Chou Chu L, Verastegui MR (2011). A history of Chagas disease transmission, control, and re-emergence in peri-rural La Joya. Peru. PLoS Negl Trop Dis..

[9] Samuels AM, Clark EH, Galdos-Cardenas G, Wiegand RE, Ferrufino L, Menacho S (2013). Epidemiology of and impact of insecticide spraying on Chagas disease in communities in the Bolivian Chaco. PLoS Negl Trop Dis..

[10] Gurtler R, Kitron U, Cecere M, Segura E, Cohen J (2007). Sustainable vector control and management of Chagas disease in the Gran Chaco. Argentina. Proc Natl Acad Sci USA..

[11] Cardinal M, Castañera M, Lauricella M, Cecere M, Ceballos L, Vazquez-Prokopec G (2006). A prospective study of the effects of sustained vector surveillance following community-wide insecticide application on *Trypanosoma cruzi* infection of dogs and cats in rural northwestern Argentina. Am J Trop Med Hyg..

[12] Cardinal MV, Lauricella MA, Marcet PL, Orozco MM, Kitron U, Gürtler RE (2007). Impact of community-based vector control on house infestation and *Trypanosoma cruzi* infection in *Triatoma infestans*, dogs and cats in the Argentine Chaco. Acta Trop..

[13] Gürtler RE, Cardinal MV (2015). Reservoir host competence and the role of domestic and commensal hosts in the transmission of *Trypanosoma cruzi*. Acta Trop..

[14] Alonso JM, Fabre AR, Galván M, Lucero RH, Brusés BL, Kuc A. La enfermedad de Chagas en poblaciones aborígenes del Noreste de Argentina. Enferm Emerg. 2009;11:115–8.

[15] Basombrío M, Segovia A, Peralta Ramos M, Esteban E, Stumpf R, Jurgensen P (1999). Endemic *Trypanosoma cruzi*, infection in Indian population of the Gran Chaco territory of South America: performance of diagnostic assays and epidemiological features. Ann Trop Med Parasitol..

[16] Biancardi MA, Conca Moreno M, Torres N, Pepe C, Altecheh J, Freilij H (2003). Seroprevalencia de la enfermedad de Chagas en 17 parajes del “Monte impenetrable” de la provincia del Chaco. Medicina (B Aires)..

[17] Lucero RH, Brusés BL, Cura CI, Formichelli LB, Juiz N, Fernández GJ (2016). Chagas’ disease in Aboriginal and Creole communities from the Gran Chaco Region of Argentina: seroprevalence and molecular parasitological characterization. Infect Genet Evol..

[18] Moretti E, Castro I, Franceschi C, Basso B (2010). Chagas disease: serological and electrocardiographic studies in Wichi and Creole communities of Misión Nueva Pompeya, Chaco, Argentina. Mem Inst Oswaldo Cruz..

[19] Sosa-Estani S, Dri L, Touris C, Abalde S, Dell’arciprete A, Braunstein J (2009). Vectorial and congenital transmission of *Trypanosoma cruzi* in Las Lomitas, Formosa. Medicina (B Aires)..

[20] Cardinal MV, Orozco MM, Enriquez GF, Ceballos LA, Gaspe MS, Alvarado-Otegui JA (2014). Heterogeneities in the ecoepidemiology of *Trypanosoma cruzi* infection in rural communities of the Argentinean Chaco. Am J Trop Med Hyg..

[21] Cohen JE, Gürtler RE (2001). Modeling household transmission of American trypanosomiasis. Science..

[22] Sartor P, Colaianni I, Cardinal MV, Bua J, Freilij H, Gürtler RE (2017). Improving access to Chagas disease diagnosis and etiologic treatment in remote rural communities of the Argentine Chaco through strengthened primary health care and broad social participation. PLoS Negl Trop Dis..

[23] Dias JCP, Silveira AC, Schofield CJ (2002). The impact of Chagas disease control in Latin America - a review. Mem Inst Oswaldo Cruz..

[24] Mott KE, Mota EA, Sherlock I, Hoff R, Muniz TM, Oliveira TS (1978). *Trypanosoma cruzi* infection in dogs and cats and household seroreactivity to *T. cruzi* in a rural community in northeast Brazil. Am J Trop Hyg..

[25] Gurevitz JM, Gaspe MS, Enriquez GF, Provecho YM, Kitron U, Gürtler RE (2013). Intensified surveillance and insecticide-based control of the Chagas disease vector *Triatoma infestans* in the Argentinean Chaco. PLoS Negl Trop Dis..

[26] Gurevitz JM, Ceballos LA, Gaspe MS, Alvarado-Otegui JA, Enríquez GF, Kitron U (2011). Factors affecting infestation by *Triatoma infestans* in a rural area of the humid Chaco in Argentina: a multi-model inference approach. PLoS Negl Trop Dis..

[27] Gurevitz JM, Gaspe MS, Enríquez GF, Vassena CV, Alvarado-Otegui JA, Provecho YM (2012). Unexpected failures to control Chagas disease vectors with pyrethroid spraying in northern Argentina. J Med Entomol..

[28] Cohen JE, Rodríguez-Planes LI, Gaspe MS, Cecere MC, Cardinal MV, Gürtler RE (2017). Chagas disease vector control and Taylor’s law. PLoS Negl Trop Dis..

[29] Brown LD, Cai TT, DasGupta A (2001). Interval estimation for a binomial proportion. Statistical Science..

[30] R Development Core Team. R: a language and environment for statistical computing. Vienna, Austria: R Foundation for Statistical Computing; 2008. https://www.r-project.org/

[31] Burnham KP, Anderson DR (2002). Model selection and multimodel inference: a practical information-theoretic approach.

[32] Bates D, Maechler M, Bolker B, Walker S (2015). Fitting linear mixed-effects models using lme4. J Stat Softw..

[33] Barton K. MuMIn: multi-model inference. 2009; http://r-forge.r-project.org/., Version 0.12.2. 2009.

[34] Lele SR, Keim JL, Solymos P. ResourceSelection: resource selection (probability) functions for use-availability Data; 2017. https://cran.r-project.org/web/packages/ResourceSelection/index.html.

[35] Hand D (2009). Measuring classifier performance: a coherent alternative to the area under the ROC curve. Mach Learn..

[36] Gürtler RE, Chuit R, Cecere MC, Castañera MB, Cohen JE, Segura EL (1998). Household prevalence of seropositivity for *Trypanosoma cruzi* in three rural villages in northwestern Argentina: environmental demographic, and entomologic associations. Am J Med Hyg..

[37] Gürtler RE, Cohen JE, Cecere MC, Lauricella MA, Chuit R, Segura EL (1998). Influence of humans and domestic animals on the household prevalence of *Trypanosoma cruzi* in *Triatoma infestans* populations in northwest Argentina. Am J Trop Med Hyg..

[38] Gaspe M, Provecho Y, Cardinal M, Fernández M, Gürtler R (2015). Ecological and sociodemographic determinants of house infestation by *Triatoma infestans* in indigenous communities of the Argentine Chaco. PLoS Negl Trop Dis..

[39] Gürtler R, Cecere M, Lauricella M, Cardinal M, Kitron U, Cohen J (2007). Domestic dogs and cats as sources of *Trypanosoma cruzi* infection in rural northwestern Argentina. Parasitology..

[40] Castillo-Neyra R, Chou Chu L, Quispe-Machaca V, Ancca-Juarez J, Malaga Chavez FS, Bastos Mazuelos M, et al. The potential of canine sentinels for reemerging *Trypanosoma cruzi* transmission. Prev Vet Med. 2015; 10.1016/j.prevetmed.2015.04.014 PMC4657134 25962956

[41] Luquetti AO, Rassi A, Brener AB-N (2000). Diagnóstico laboratorial da infecção pelo *Trypanosoma cruzi*. *Trypanosoma cruzi* e doença de Chagas. 2nd edn. Rio de Janeiro, Brasil: Editorial Guanabara Koogan SA.

[42] Hoff R, Mott KE, França Silva J, Menezes V, Hoff JN, Barrett TV (1979). Prevalence of parasitemia and seroreactivity to *Trypanosoma cruzi* in a rural population of northeast Brazil. Am J Trop Med Hyg..

[43] Borges Pereira J, Faraco Willcox HP, Brisola Marcondes C, Rodrigues Coura J (1989). Parasitemia em pacientes chagásicos crônicos avaliada pelo índice de triatomíneos infectados no xenodiagnóstico. Rev Soc Bras Med Trop..

[44] Gürtler RE, Cecere MC, Vázquez-Prokopec GM, Ceballos LA, Gurevitz JM, Fernández MDP (2014). Domestic animal hosts strongly influence human-feeding rates of the Chagas disease vector *Triatoma infestans* in Argentina. PLoS Negl Trop Dis..

[45] Aiga H, Sasagawa E, Hashimoto K, Nakamura J, Zúniga C, Chévez JER (2012). Chagas disease: assessing the existence of a threshold for bug infestation rate. Am J Trop Med Hyg..

[46] Abad-Franch F, Valença-Barbosa C, Sarquis O, Lima MM (2014). All that glisters is not gold: sampling-process uncertainty in disease-vector surveys with false-negative and false-positive detections. PLoS Negl Trop Dis..

[47] Zu Dohna H, Cecere MC, Gürtler RE, Kitron U, Cohen JE (2007). Re-establishment of local populations of vectors of Chagas disease after insecticide spraying. J Appl Ecol..

[48] Gürtler RE, Cecere MC, Canale DM, Castañera MB, Chuit R, Cohen JE (1999). Monitoring house reinfestation by vectors of Chagas disease: a comparative trial of detection methods during a four-year follow-up. Acta Trop..

[49] Segura E, Silveira A (2002). El control de la enfermedad de Chagas en la República Argentina. El control de la enfermedad de Chagas en los países del Cono Sur de América. Historia de una iniciativa internacional 1991/2001.

[50] Blanco S, Segura E, Gürtler R (1999). El control de la transmisión congénita de *Trypanosoma cruzi* en la Argentina. Med (Buenos Aires)..

[51] Blanco EL, Cura EN, Chuit R, Tulián L, Flores I, Garbarino G, Villalonga F, Gurtler RE (2000). Congenital transmission of *Trypanosoma cruzi*: an operational outline for detecting and treating infected infants in north-western Argentina. Trop Med Int Heal.

[52] Sánchez Negrette O, Mora M, Basombrío M (2005). High Prevalence of congenital *Trypanosoma cruzi* infection and family clustering in Salta, Argentina. Pediatrics.

[53] Enriquez GF, Bua J, Orozco MM, Wirth S, Schijman AG, Gürtler RE, Cardinal MV (2014). High levels of *Trypanosoma cruzi* DNA determined by qPCR and infectiousness to *Triatoma infestans* support dogs, and cats are major sources of parasites for domestic transmission. Infect Genet Evol..

