# Supplementary material for: High levels of human infection with Trypanosoma cruzi associated with the domestic density of infected vectors and hosts in a rural area of northeastern Argentina
Source: Parasit Vectors. 2018 Aug 30;11:492. doi: 10.1186/s13071-018-3069-0 (PMC6118006; doi:10.1186/s13071-018-3069-0)
Supplement: Supplementary file 1 — Timeline of key events. (PDF 203 kb) [file 13071_2018_3069_MOESM1_ESM.pdf]

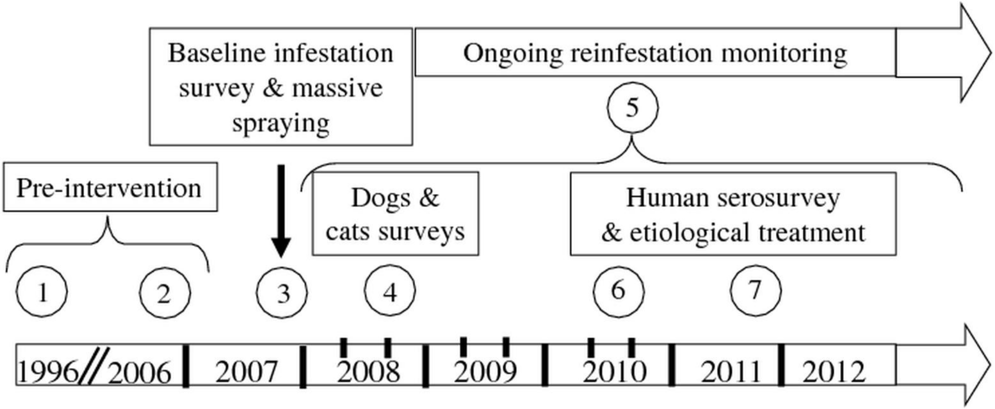

1: Last community-wide insecticide spraying campaign conducted by vector control program staff.

2: Isolated insecticide treatments applied by villagers or hospital staff.

3: Baseline cross-sectional entomological survey of all houses conducted in October-November 2007. Triatoma infestans were collected in 39.8% of inhabited house compounds by timed manual collections with a dislodging spray. [26-27]

Vector control personnel sprayed all house structures with suspension concentrate deltamethrin (K-Othrin, Bayer) at standard dose (25 mg/m2) in November-December 2007.[25-26]

4: Three non-overlapping, cross-sectional, house-to-house surveys targeting all dogs and cats residing in 7 contiguous villages totaling 173 inhabited houses in August, September and December 2008.[20]

5: All houses were re-inspected every 4-7 months postspraying during the first three years and annually thereafter. Infested houses were re-sprayed at each survey.[25]

6: Human serosurvey.

7: Etiological treatments.[20]
