# Supplementary material for: High levels of human infection with Trypanosoma cruzi associated with the domestic density of infected vectors and hosts in a rural area of northeastern Argentina
Source: Parasit Vectors. 2018 Aug 30;11:492. doi: 10.1186/s13071-018-3069-0 (PMC6118006; doi:10.1186/s13071-018-3069-0)
Supplement: Supplementary file 2 — Table S1. STROBE Statement - checklist of items that should be included in reports of cross-sectional studies. Table S2. Comparison among serological test results for Trypanosoma cruzi infection in Area I human residents of Pampa del Indio, Chaco. Table S3. Model-averaged coefficients of factors associated with Trypanosoma cruzi infection in humans born before the intervention programme in Pampa del Indio, Chaco. (DOCX 33 kb) [file 13071_2018_3069_MOESM2_ESM.docx]

**Additional file 2. Table S1.** STROBE Statement - checklist of items that should be included in reports of cross-sectional studies.

|  | Item No | Recommendation | Lines | |  |
| --- | --- | --- | --- | --- | --- |
| **Title and abstract** | 1 | (*a*) Indicate the study’s design with a commonly used term in the title or the abstract | 26-29 | |  |
|  |  | (*b*) Provide in the abstract an informative and balanced summary of what was done and what was found | 26-46 | |  |
| Introduction | | |  | |  |
| Background/rationale | 2 | Explain the scientific background and rationale for the investigation being reported | 57-100 | |  |
| Objectives | 3 | State specific objectives, including any prespecified hypotheses | 83-86;  87-100 | |  |
| Methods | | |  | |  |
| Study design | 4 | Present key elements of study design early in the paper | 115-119 | |  |
| Setting | 5 | Describe the setting, locations, and relevant dates, including periods of recruitment, exposure, follow-up, and data collection | Additional File 1,  103-114; 115-139 | |  |
| Participants | 6 | (*a*) Give the eligibility criteria, and the sources and methods of selection of participants | 115-119;  134-136 | |  |
| Variables | 7 | Clearly define all outcomes, exposures, predictors, potential confounders, and effect modifiers. Give diagnostic criteria, if applicable | 142-145; 149-150; 153-155; 180-187. | |  |
| Data sources/ measurement | 8* | For each variable of interest, give sources of data and details of methods of assessment (measurement). Describe comparability of assessment methods if there is more than one group | 120-133; 142-156; | |  |
| Bias | 9 | Describe any efforts to address potential sources of bias | 107-109  220-222 | |  |
| Study size | 10 | Explain how the study size was arrived at | 157-160  196-201  220-226 | |  |
| Quantitative variables | 11 | Explain how quantitative variables were handled in the analyses. If applicable, describe which groupings were chosen and why | 158-161  185-195 | |  |
| Statistical methods | 12 | (*a*) Describe all statistical methods, including those used to control for confounding | 154-217 | |  |
|  |  | (*b*) Describe any methods used to examine subgroups and interactions | 201-203 | |  |
|  |  | (*c*) Explain how missing data were addressed | 161-162;  196-201 | |  |
|  |  | (*d*) If applicable, describe analytical methods taking account of sampling strategy |  | |  |
|  |  | (*e*) Describe any sensitivity analyses | 174-179 | |  |
| Results | | |  | |  |
| Participants | 13* | (a) Report numbers of individuals at each stage of study—eg numbers potentially eligible, examined for eligibility, confirmed eligible, included in the study, completing follow-up, and analysed | 157-160  196-201  220-225 | |  |
|  |  | (b) Give reasons for non-participation at each stage | 223-226 | |  |
|  |  | (c) Consider use of a flow diagram |  | |  |
| Descriptive data | 14* | (a) Give characteristics of study participants (eg demographic, clinical, social) and information on exposures and potential confounders | 231-236;  237-247; 257-283; Table 1, 2, 3 | |  |
|  |  | (b) Indicate number of participants with missing data for each variable of interest | In M&M | |  |
| Outcome data | 15* | Report numbers of outcome events or summary measures | 226  Table1 | |  |
| Main results | 16 | (*a*) Give unadjusted estimates and, if applicable, confounder-adjusted estimates and their precision (eg, 95% confidence interval). Make clear which confounders were adjusted for and why they were included | | 248-249; 267-298; Table 4 | |
|  |  | (*b*) Report category boundaries when continuous variables were categorized | | Table 4 | |
|  |  | (*c*) If relevant, consider translating estimates of relative risk into absolute risk for a meaningful time period | |  | |
| Other analyses | 17 | Report other analyses done—eg analyses of subgroups and interactions, and sensitivity analyses | | 252-256  267-282;  Table 5 | |
| Discussion | | | |  | |
| Key results | 18 | Summarise key results with reference to study objectives | | 301-312; 448-452. | |
| Limitations | 19 | Discuss limitations of the study, taking into account sources of potential bias or imprecision. Discuss both direction and magnitude of any potential bias | | 411-430 | |
| Interpretation | 20 | Give a cautious overall interpretation of results considering objectives, limitations, multiplicity of analyses, results from similar studies, and other relevant evidence | | 431-445 | |
| Generalisability | 21 | Discuss the generalisability (external validity) of the study results | | 312-317 | |
| Other information | | | |  | |
| Funding | 22 | Give the source of funding and the role of the funders for the present study and, if applicable, for the original study on which the present article is based | | 465-468 | |

*Give information separately for exposed and unexposed groups.

**Additional file 2: Table S2**. Comparison among serological test results for *Trypanosoma cruzi* infection in Area I human residents of Pampa del Indio, Chaco.

|  | ELISA (lysate, recombinant) test result | | | | | |
| --- | --- | --- | --- | --- | --- | --- |
| IFAT ^a^ | | +, + | +,- | -,+ | -,- | Total |
| Positive | | 14 | 0 | 14 | 0 | 28 |
| Negative | | 0 | 0 | 5 | 10 | 15 |
| Not done | | 252 | 0 | 0 | 420 | 672 |
| Total | | 266 | 0 | 19 | 430 | 715 |

^a^ IFAT was mainly used to test ELISA-discordant sera.

| **Additional file 2: Table S3.** Model-averaged coefficients of factors associated with *Trypanosoma cruzi* infection in humans, Pampa del Indio. | | | | | | |
| --- | --- | --- | --- | --- | --- | --- |
|  |  |  |  |  | 95% CI | |
| Variable^a^ | Coefficient | S.E. | *Z* | *P* | Low | High |
| Intercept | -0.741 | 0.174 | 4.255 | 0.000 | -1.083 | -0.400 |
| Born after the onset of the intervention program | -2.233 | 0.805 | 2.773 | 0.005 | -3.811 | -0.655 |
| Age (in years) | 0.931 | 0.118 | 7.884 | 0.000 | 0.700 | 1.163 |
| Infected-bug abundance | |  |  |  |  |  |
| No infected bugs | 0.161 | 0.263 | 0.614 | 0.540 | -0.354 | 0.677 |
| ≥1 infected bugs | 1.059 | 0.275 | 3.855 | 0.000 | 0.521 | 1.598 |
| Number of infected cohabitants | |  |  |  |  |  |
|  | 0.830 | 0.123 | 6.753 | 0.000 | 0.589 | 1.071 |
| Ethnic group |  |  |  |  |  |  |
| Qom | -0.423 | 0.258 | 1.639 | 0.101 | -0.930 | 0.083 |
| Gender |  |  |  |  |  |  |
| Male | 0.136 | 0.204 | 0.664 | 0.506 | -0.265 | 0.536 |
| Goat-equivalent index | -0.115 | 0.105 | 1.102 | 0.270 | -0.321 | 0.090 |
| ^a^ Variables (see text for details): Born after the onset of the intervention program, 2 levels, age (in years); infected-bug abundance, 3 levels; number of infected cohabitants; ethnic group, (Qom or Creole); gender, 2 levels; goat-equivalent index per 10 goats. Age, goat equivalent index and number of infected cohabitants were standardized. | | | | | | |
| Reference levels were born before the onset of the intervention program, the average age, no observed infestation at the household, the average number of infected cohabitants, females, Creoles, and the average goat equivalent index. | | | | | | |
